# Animal models for assessing hypoallergenic clinical performance potential of products based on hydrolyzed protein systems

**DOI:** 10.1186/2045-7022-1-S1-O17

**Published:** 2011-08-12

**Authors:** Christopher Cordle, Geralyn Duska-McEwen, Ricardo Rueda, Enrique Vazquez

**Affiliations:** 1 Abbott Nutrition, Columbus, USA; 2 Abbott Nutrition, Granada, Spain

## 

Formulas based on protein hydrolysates or free amino acids are used to manage food allergies in infants and young children. Three formula categories are available: Products containing partially hydrolyzed proteins which show some decrease in clinical reactivity but are not recommended for food-allergic infants, formulas based on extensively hydrolyzed proteins that are useful in clinical management of food allergies in >90% of patients, and free amino acid-based formulas used for the most strongly allergic patients.

Formula hypoallergenic reactivity is only established by clinical trials, but these studies should be preceded by evaluation in appropriate animal models. For partially hydrolyzed formulas the goal of the model is to demonstrate some reduction in immunologic reactivity that justifies their "Hypoallergenic, not for use in food-allergic infants" labeling. For formulas based on extensively hydrolyzed proteins or amino acids, results of the animal models must predict the accepted standard for hypoallergenic clinical performance (Double-blinded, placebo-controlled food challenges indicating 90% of allergic patients tolerate the formula, 95% CI).

Guinea pig oral sensitization is used to show decreased immunological reactivity of partially hydrolyzed formulas. Model sensitivity can be controlled by varying feeding duration, with positive controls (intact cow milk formula) reacting after a 15 day feeding. However, some partially hydrolyzed formulae that give negative results with 15 day feedings are sensitizing if fed for 35 days. Extensively hydrolyzed and amino acid formulas fed 35 days are not sensitizing. Symptom standards for evaluating reactions will be presented along with formula type and feeding duration comparisons.

The laboratory animal hyperimmunization model is sensitive at the lower end of the immunological reactivity scale and can be used to differentiate extensively hydrolyzed and amino acid formulas which meet hypoallergenic clinical performance standards from partially hydrolyzed formulas not recommended for allergic patients. Analytical considerations and formula type comparisons will be presented.

